# Morphological analysis of the distal femur as a surgical reference in biplane distal femoral osteotomy

**DOI:** 10.1038/s41598-024-62988-y

**Published:** 2024-05-27

**Authors:** Shohei Sano, Takehiko Matsushita, Naosuke Nagata, Takeo Tokura, Koji Nukuto, Yuta Nakanishi, Kyohei Nishida, Kanto Nagai, Noriyuki Kanzaki, Yuichi Hoshino, Tomoyuki Matsumoto, Ryosuke Kuroda

**Affiliations:** Department of Orthopaedic Surgery, Kobe University Graduate School of Medicine, 7-5-1 Kusunoki-cho, Chuo-ku, Kobe, 650-0017 Japan

**Keywords:** Musculoskeletal system, Bone imaging

## Abstract

Distal femoral osteotomy (DFO) is performed alone or with high tibial osteotomy (HTO) for patients with osteoarthritis and distal femur deformities. DFO is technically demanding, particularly when creating an anterior flange. Herein, we examined the morphological characteristics of the distal femur based on the cortical shape as a surgical reference for biplanar DFO. Computed tomography images of 50 valgus and 50 varus knees of patients who underwent biplanar DFO or total knee arthroplasty were analyzed. Axial slices at the initial level of the transverse osteotomy in the DFO and slices 10 mm proximal and 10 mm distal to that level were selected. The medial and lateral cortical angles and heights (MCLA, LCLA, MCH, and LCH) were measured on axial slices. Statistical comparisons were performed between the medial and lateral cortices and valgus and varus knees. MCLA and MCH were significantly smaller and lower, respectively, than LCLA and LCH (*P* < 0.01). The MCLA and MCH of varus knees were significantly smaller and lower, respectively, than those of valgus knees (*P* < 0.01). Surgeons should carefully observe morphological differences in the distal femur cortex, distinguishing between medial and lateral knees and varus and valgus knees during the creation of the anterior flange in the DFO.

## Introduction

Distal femoral osteotomy (DFO) is a surgical procedure used alone or in combination with proximal tibial osteotomy to treat patients with lower-limb malalignment^1–9^. Medial closing wedge DFO (MCWDFO) is performed in patients with valgus knee osteoarthritis (OA)^3,5,8,10–13^, whereas lateral closing wedge DFO (LCWDFO) is performed in conjunction with high tibial osteotomy (HTO) to treat severe varus knee OA, creating a double-level osteotomy (DLO)^14–19^.

**Figure 1:**
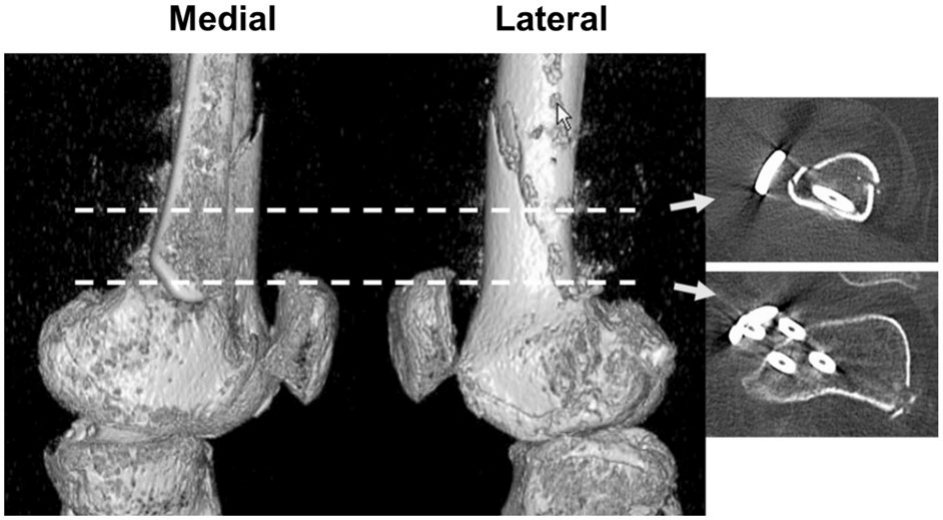
A case of biplanar medial closing wedge osteotomy (MCWDFO). The patient is a 36-year-old female. (Left) Lateral and medial view images of three-dimensional computed tomography, 2 weeks after surgery. The oblique osteotomy line seen on the image from the lateral side corresponds to the osteotomy line of the anterior flange. (Right) Axial images correspond to the level indicated by the broken lines.

Regarding surgical techniques, biplanar osteotomy has gained popularity in HTO and DFO. Its widespread adoption is attributed to its advantages, such as a large contact area and better stability against axial load^20,21^. In addition, the biplanar osteotomy technique offers the advantage of effectively reducing fragments in hinge fractures. The anterior flange can be used to control fragment rotation during the procedure. Although the biplanar osteotomy technique offers such advantages, it is more technically challenging than single-plane osteotomy. In biplanar DFO, careful attention is required to determine the osteotomy angle and thickness when creating the anterior flange. A higher incidence of hinge fractures occurs after MCWDFO than after LCWDFO via DLO^22^. Clinical experience has shown a higher occurrence of hinge fractures in patients who underwent MCWDFO than in those who underwent LCWDFO due to an excessively thick anterior flange This resulted in a substantial decrease in the width of the opposite hinge compared to those who underwent LCWDFO (Fig. 1). This difference appears to be attributable to anatomical differences between the medial and lateral cortices. Familiarity with the anatomical shape of the distal femur is crucial for accurately forming the anterior flange in biplanar DFO. However, details of the distal femoral bony morphology for creation of anterior flange in distal femoral osteotomy have not been reported.

This study aimed to examine the morphological characteristics of the bony cortex of the distal femur using axial view slices of computed tomography of valgus and varus knees. We hypothesized that the distal femoral cortex is shorter and more gently inclined against the posterior femoral cortex in axial view at the osteotomy site on the medial side than on the lateral side and that the distal femoral cortex of valgus knees is smaller and more gently inclined than that of varus knees.

## Materials and methods

### Patients

In total, 100 knees of 97 patients who underwent DFO or total knee arthroplasty (TKA) between February 2018 and March 2022 at our institution were included in this study. To analyze valgus knee OA, 50 knees (primary OA: 39 knees, post-surgical or traumatic OA: 11 knees) of 49 patients who underwent TKA or DFO for this condition were selected. To analyze varus knees, 50 knees (primary OA: 40 knees, post-surgical or traumatic OA: 10 knees) from 48 patients who underwent HTO or DLO for this condition were selected (Table 1). This retrospective study was approved by the Institutional Review Board of Kobe University Graduate School of Medicine, and written informed consent was obtained from all patients before their enrollment. All methods were performed in accordance with the relevant guidelines and regulations.

**Table 1:** Patient demographic, surgical, and radiographic data.

	Valgus (n = 50)	Varus (n = 50)	*P* value
Patient demographics			
Age (years)	66.0 ± 16.5	60.5 ± 6.9	< 0.01
Gender (male/female)	16/34	30/20	< 0.01
Height (cm)	157.7 ± 10.3	163.9 ± 8.0	< 0.01
Weight (kg)	61.9 ± 12.4	70.4 ± 11.2	< 0.01
BMI (kg/m^2^)	24.8 ± 3.5	26.2 ± 3.6	< 0.05
Radiographic evaluation			
Preoperative FTA (°)	165.8 ± 7.2	180.4 ± 2.8	< 0.01
Preoperative %MA (%)	88.9 ± 30.3	20.5 ± 13.6	< 0.01
Evaluation of surgical procedures			
TKA/Osteotomy (n)	35/15	0/50	< 0.01

*n,* number of knees; *FTA,* femorotibial angle; *MA,* mechanical axis; *n.s,* not significant.

### Inclusion and exclusion criteria

The inclusion criteria were as follows: (1) patients who underwent TKA or knee osteotomy (DFO, HTO, DLO) and (2) those who underwent plain computed tomography prior to surgery. The exclusion criteria were as follows: (1) severe post-traumatic deformity, (2) post-infectious knee arthritis, and (3) rheumatoid arthritis.

### Surgical indication

The indications for MCWDFO were lateral compartment OA with cartilage damage, patellar dislocation with a mechanical lateral distal femoral angle (mLDFA) ≤ 85°, and % mechanical axis (MA) ≥ 55%; HTO was recommended for patients with medial knee OA. DLO was performed if a medial opening gap > 20 mm was required or if the mechanical proximal angle exceeded 95° after correction to achieve the target alignment in preoperative planning. DLO was also performed in patients with an mLDFA ≥ 90°. Indications for TKA for valgus knee OA are low activity levels and severe knee deformities.

### Measurements

#### Measurement level

Preoperative axial CT images taken for surgical planning were used for these measurements. An axial slice 65 mm proximal to the knee joint line, which corresponds to 40 mm proximal to the medial or lateral epicondyle, served as the primary reference level. The slice position was selected based on the average starting level of the transverse osteotomy in patients who underwent DFO at our hospital. Subsequently, the cortex angle and height were measured on the reference slice, along with slices positioned 10 mm proximal and 10 mm distal to the reference slice.

#### Angle

**Figure 2:**
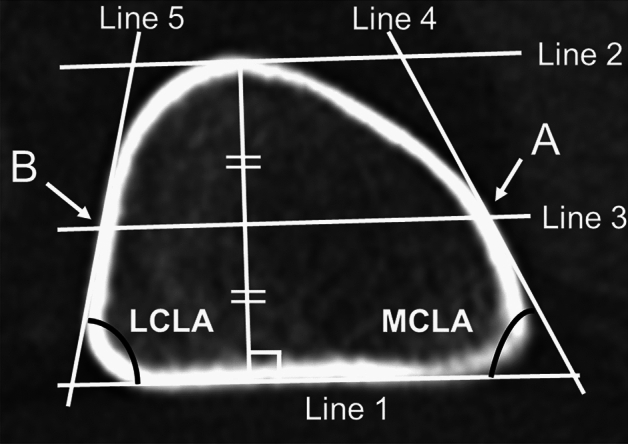
Measurement of the femoral cortex angle. An image of the axial view of the right knee at the reference level.

First, a straight line was drawn along the posterior margin of the femur (Line 1). Second, we drew a line parallel to Line 1 through the front tip of the femur (Line 2). Third, we drew a line between the middle of Lines 1 and 2 (Line 3) and determined the intersection of the inside and outside (A, B). Fourth, tangential lines were drawn through Point A to the medial cortex (Line 4) and through Point B to the lateral cortex (Line 5). Fifth, the angle between Line 1 and Line 4 was defined as the medial cortex line angle (MCLA), and the angle between Line 1 and Line 5 was termed the lateral cortex line angle (LCLA) (Fig. 2).

#### Height

**Figure 3:**
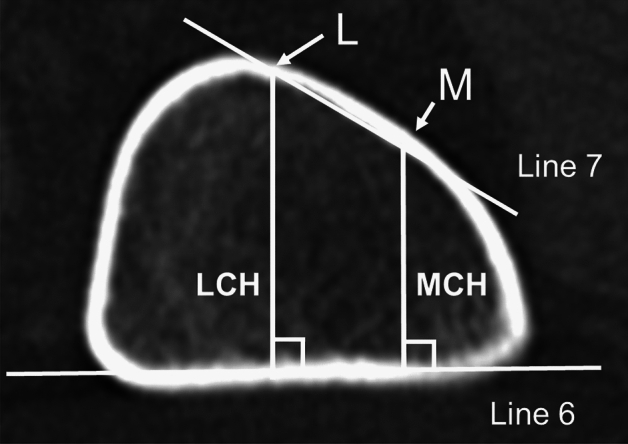
Measurement of the femoral cortex height.

First, tangential lines were drawn to the inner margin of the posterior cortex (Line 6) and anterior cortex (Line 7). Second, a vertical line was drawn from Lines 6 to 7, with the medial intersection point as Point M and the lateral intersection point as Point L. Third, the vertical distance between Line 6 and Point M was defined as the medial cortex height (MCH), and that between Line 7 and Point L was defined as the lateral cortex height (LCH) (Fig. 3).

### Statistical analyses

The number of samples was determined based on a priori sample size calculations in a preliminary study using randomly selected 10 valgus and 10 varus knees. A priori power analysis using G*Power (Heinrich Heine Universitȁt Dȕsseldorf, Germany) indicated a minimum of 47 samples for each group to detect a difference between the two groups with an alpha level of 0.05, a beta of 80%, and an effect size of 0.58.

Considering the error margin, the sample size was set to 50 for each group. For the comparison between varus and valgus knees, comparison was performed including all the patients and patients matched with age, gender, height, weight, and BMI. All statistical analyses were performed using EZR (Saitama Medical Center, Jichi Medical University, Saitama, Japan), a graphical user interface for R (R Foundation for Statistical Computing, Vienna, Austria)^23^. The normality test was the Shapiro–Wilk test. Owing to the normality of the data, the Mann–Whitney U test or Student t-test was used to compare continuous values between the two groups. Fisher’s exact test was used to compare categorical values. Statistical significance was set at *P* < 0.05. The inter-class correlation coefficient (ICC) was used to assess inter-rater and intra-rater reliability for MCLA, LCLA, MCH, and LCH. ICC < 0.50 was considered poor reliability, 0.50 ≤ ICC < 0.75 was considered moderate reliability, 0.75 ≤ ICC < 0.90 was considered good reliability, and ICC ≥ 0.90 was considered excellent reliability^24^.

## Results

### Validation of measurement methods for distal femur shape

The ICC values for each angle and height (MCLA, LCLA, MHC, and LCH) are summarized in Supplemental Table 1. Overall, good-to-excellent intra-rater reliability was obtained for MCLA, LCLA, MHC, and LCH. Similarly, good-to-excellent ICC values for inter-rater agreement were obtained (Supplemental Table 1).

### Comparison of the cortex angle between the medial and lateral side of the distal femur in valgus and varus knees

The mean MCLA was significantly smaller than the LCLA in both valgus and varus knees at all three levels (*P* < 0.01) (Table. 2).

**Table 2:** Comparison between MCLA and LCLA in valgus and varus knees.

	MCLA (°)	LCLA (°)	*P* value
Valgus			
10 mm proximal	69.2 ± 8.5	79.1 ± 4.8	< 0.01
Reference	68.1 ± 8.5	78.4 ± 4.3	< 0.01
10 mm distal	67.0 ± 7.5	79.5 ± 4.4	< 0.01
Varus			
10 mm proximal	75.2 ± 6.7	81.3 ± 4.1	< 0.01
Reference	74.8 ± 5.9	80.4 ± 4.7	< 0.01
10 mm distal	71.8 ± 5.3	81.2 ± 4.5	< 0.01

Values are the mean ± standard deviation.

### Comparison of cortex height between the medial and lateral side of the distal femur in *valgus* and varus knees

The mean LCH was significantly higher than the mean MCH in the valgus and varus knees at all three levels (*P* < 0.01) (Table 3).

**Table 3:** Comparison between MCH and LCH in valgus and varus knees.

	MCH (mm)	LCH (mm)	*P* value
Valgus			
10 mm proximal	20.4 ± 3.5	26.4 ± 3.6	< 0.01
Reference	21.3 ± 3.5	27.3 ± 3.8	< 0.01
10 mm distal	23.5 ± 4.0	29.5 ± 4.5	< 0.01
Varus			
10 mm proximal	23.7 ± 3.1	29.4 ± 3.3	< 0.01
Reference	24.7 ± 3.1	30.3 ± 3.3	< 0.01
10 mm distal	27.1 ± 3.4	32.7 ± 3.5	< 0.01

Values are the mean ± standard deviation.

### Comparison of cortex angle and height between *valgus* and varus knees

The mean MCLA in valgus knees was significantly smaller than that in varus knees at all three levels (*P* < 0.01). The mean LCLA in valgus knees was significantly smaller than that in varus knees at the 10 mm proximal (*P* = 0.013) and reference levels (*P* = 0.015). No significant difference was observed between valgus and varus knees at the 10 mm distal level. The mean MCH and LCH in valgus knees were significantly lower than those in varus knees at all three levels (*P* < 0.01) (Table 4).

**Table 4:** Comparison between valgus and varus knees in MCLA, LCLA, MCH, and LCH.

	Valgus	Varus	*P* value
MCLA (°)			
10 mm proximal	69.2 ± 8.5	75.2 ± 6.7	< 0.01
Reference	68.1 ± 8.5	74.8 ± 5.9	< 0.01
10 mm distal	67.0 ± 7.5	71.8 ± 5.3	< 0.01
LCLA (°)			
10 mm proximal	79.1 ± 4.8	81.3 ± 4.1	0.013
Reference	78.4 ± 4.3	80.4 ± 4.7	0.015
10 mm distal	79.5 ± 4.4	81.2 ± 4.5	n.s
MCH (mm)			
10 mm proximal	20.4 ± 3.5	23.7 ± 3.1	< 0.01
Reference	21.3 ± 3.5	24.7 ± 3.1	< 0.01
10 mm distal	23.5 ± 4.0	27.1 ± 3.4	< 0.01
LCH (mm)			
10 mm proximal	26.4 ± 3.6	29.4 ± 3.3	< 0.01
Reference	27.3 ± 3.8	30.3 ± 3.3	< 0.01
10 mm distal	29.5 ± 4.5	32.7 ± 3.5	< 0.01

Values are the mean ± standard deviation.

## Discussion

The primary discovery of this study was the shorter and more inclined nature of the medial cortex compared to the lateral cortex of the distal femur. In addition, the distal femoral cortex of valgus knees was smaller and more inclined than that of varus knees on both the medial and lateral sides.

Several previous reports have described surgical techniques for ascending osteotomy of the anterior flange in biplanar DFO, focusing on the angle against transverse osteotomy and flange length. Previous reports have described initiating the ascending cut at the anterior one-fourth of the femur, followed by creating the anterior flange at an angle of 90–95° relative to the transverse osteotomy, with a length of 3–4 cm in the biplanar MCWDFO^5,8,20,25–28^. Similarly, Woude et al.^21^ reported that the ascending osteotomy was initiated at the anterior one-fourth of the distal femur and directed proximally in biplanar LCWDFO. Although these reports described the angle against transverse osteotomy, previous studies did not specifically address the angle against the femoral cortex in the axial plane. In this study, significant differences between the shapes of the medial and lateral cortex and valgus and varus knees were revealed. In the reference slice where the transverse osteotomy was initiated, the mean MCLA of the valgus knee was 68.1°, whereas that of the varus knee was 80.4°. Regarding cortex height, the mean MCH of valgus knees was 21.3 mm, whereas that of varus knees was 30.3 mm. Considering that the width of the shaft of the DFO plates was approximately 20 mm, less than 10 mm was left for the anterior flange in the biplanar medial DFO. Thus, it is necessary to carefully determine the anterior flange thickness during biplanar medial DFO, particularly in patients with small femurs. Although the appropriate flange thickness and angle must be determined, our results may provide valuable information for determining the cutting angle and starting point for creating the surgeon’s preferred anterior flange.

**Figure 4:**
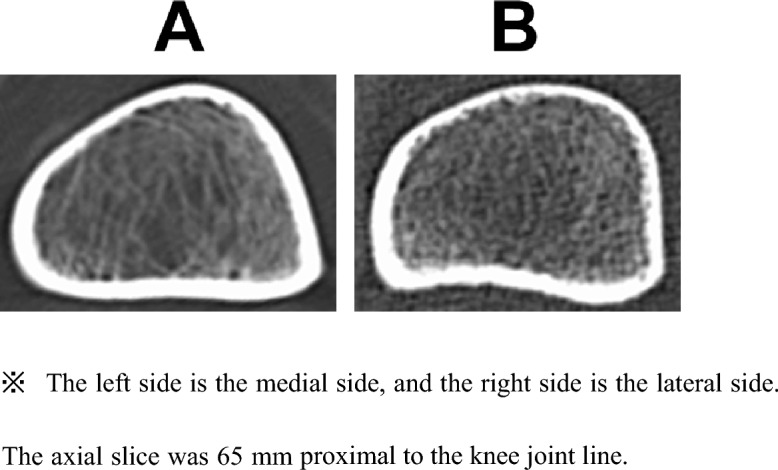
Typical femur shape in valgus and varus knee. (**A**) 56-year-old female with a valgus knee (Left knee). (**B**) 56-year-old female with a varus knee (Left knee).

In this study, a flattened shape was observed in the medial cortex of some patients with valgus knees. Differentiating the turning point between the anterior and medial cortices was challenging in these cases. Therefore, surgeons may make errors when choosing the appropriate height and angle for creating the anterior flange during biplanar MCWDFO. Given that vertical cuts are easier than oblique cuts into the cortex, surgeons tend to cut vertically into the cortex when creating the anterior flange (Fig. 4). In such cases, the width of the opposite hinge is small, particularly in the MCWDFO. Therefore, careful attention is required when creating an anterior flange, particularly for biplanar MCWDFO. Fujita et al.^29^ reported hinge fractures in 57% of patients after biplanar MCWDFO. Matsushita et al.^22^ reported a significantly higher incidence of hinge fractures after biplanar MCWDFO than after LCWDFO via DLO (70% vs. 30%). These reports showed a high incidence of hinge fractures in biplanar MCWDFO. The results of the present study suggest that an inappropriate anterior flange may be partially associated with a high incidence of hinge fractures in MCWDFO. Kim et al.^30^ reported a 42% incidence of hinge fractures in patients undergoing various types of DFO, including MCWDFO, LCWDFO, medial opening wedge DFO, and lateral opening wedge DFO. Notably, they observed that the anterior flange thickness was not associated with hinge fractures. However, their measurement of anterior flange thickness focused solely on the osteotomy side in the sagittal view, excluding the opposite hinge side. Further studies are required to determine the involvement of the anterior flange in hinge fractures.

### Limitations

This study has some limitations. First, the number of patients, particularly those who underwent osteotomy, was small; however, the study confirmed sufficient power to detect differences between the groups. Additionally, the current study focused solely on the differences between varus and valgus knees, neglecting surgical indications, and the number of patients unlikely to affect the results. Second, patients with valgus knees were significantly older and shorter than those with varus knees. This significant difference might be influenced by variations in generation and body size. However, almost similar results were found when comparing 33 knees each within the varus and valgus knee groups, where there were no significant differences in demographic data (Supplemental Table 2). Therefore, the result of this study can be a useful information for DFO. Our study suggests that surgeons should be careful when performing biplanar DFO in older patients with valgus knees. Third, the study did not examine the influence of the anterior flange on the incidence of hinge fractures in biplanar DFO. Therefore, future research should focus on determining suitable cutting angles and anterior flange thicknesses. Fouth, although a consistent reference level was used in all the knees, the reference level may not be the starting level of the transverse osteotomy for each patient Therefore, the results can be different if the measurement was performed choosing different slice level. However, the reference level was similar to the transverse osteotomy starting level in previous studies^31,32^. In addition, 10 mm proximal and distal slices were also evaluated in this study to cover the range of the widely used osteotomy levels. Therefore, our results can be useful. Despite these limitations, this study provides useful information regarding the anatomical characteristics of the distal femur in biplanar DFO.

## Conclusion

At the osteotomy level, the medial cortical angle and height were shorter and more inclined than those of the lateral cortex in the distal femurs, especially in patients with valgus knees. These results suggest that careful attention is required when creating an anterior flange during biplanar DFO, particularly in patients with valgus knees.

### Supplementary Information

Supplementary Information.

## Data Availability

Data supporting the findings of this study are available upon request from the corresponding authors. The data are not publicly available due to privacy concerns.
