# Supplementary material for: Morphological analysis of the distal femur as a surgical reference in biplane distal femoral osteotomy
Source: Sci Rep. 2024 May 27;14:12130. doi: 10.1038/s41598-024-62988-y (PMC11130220; doi:10.1038/s41598-024-62988-y)
Supplement: Supplementary file 1 — Supplementary Information. [file 41598_2024_62988_MOESM1_ESM.doc]

**Supplemental Table 1.**

Interclass correlation coefficient values for MCLA (medial cortex line angle), LCLA (lateral cortex line angle), MCH (medial cortex height), LCH (lateral cortex height) of valgus and varus knees.

**Valgus knee**

**Intra-rater**

|  | **MCLA** | **LCLA** | **MCH** | **LCH** |
| --- | --- | --- | --- | --- |
| **Reference** | **0.86 (0.56–0.96)** | **0.82 (0.45–0.95)** | **0.89 (0.63–0.97)** | **0.86 (0.57–0.96)** |
| **10 mm proximal** | **0.85 (0.52–0.96)** | **0.8 (0.4–0.94)** | **0.84 (0.51–0.96)** | **0.91 (0.71–0.98)** |
| **10 mm distal** | **0.93 (0.75–0.98)** | **0.83 (0.48–0.95)** | **0.86 (0.55–0.96)** | **0.88 (0.61–0.97)** |

**Inter-rater**

|  | **MCLA** | **LCLA** | **MCH** | **LCH** |
| --- | --- | --- | --- | --- |
| **Reference** | **0.80 (0.41–0.95)** | **0.81 (0.39–0.95)** | **0.85 (0.4–0.96)** | **0.78 (0.26–0.94)** |
| **10 mm proximal** | **0.87 (0.12–0.97)** | **0.76 (0.33–0.93)** | **0.82 (0.41–0.95)** | **0.82 (0–0.97)** |
| **10 mm distal** | **0.86 (0.53–0.96)** | **0.82 (0.46–0.95)** | **0.88 (0.6–0.97)** | **0.85 (0.12–0.97)** |

**Varus knee**

**Intra-rater**

|  | **MCLA** | **LCLA** | **MCH** | **LCH** |
| --- | --- | --- | --- | --- |
| **Reference** | **0.84 (0.51–0.96)** | **0.89 (0.64–0.97)** | **0.86 (0.56–0.96)** | **0.8 (0.4–0.94)** |
| **10 mm proximal** | **0.82 (0.47–0.95)** | **0.85 (0.52–0.96)** | **0.86 (0.55–0.96)** | **0.87 (0.6–0.97)** |
| **10 mm distal** | **0.96 (0.87–0.99)** | **0.86 (0.56–0.96)** | **0.87 (0.57–0.96)** | **0.88 (0.16–0.98)** |

**Inter-rater**

|  | **MCLA** | **LCLA** | **MCH** | **LCH** |
| --- | --- | --- | --- | --- |
| **Reference** | **0.86 (0.19–0.97)** | **0.89 (0.62–0.97)** | **0.85 (0.06–0.97)** | **0.78 (0.3–0.94)** |
| **10 mm proximal** | **0.80 (0.36–0.95)** | **0.8 (0.37–0.95)** | **0.82 (0.42–0.95)** | **0.91 (0.68–0.98)** |
| **10 mm distal** | **0.96 (0.83–0.99)** | **0.89 (0.61–0.97)** | **0.88 (0.16–0.98)** | **0.87 (0.3–0.97)** |

**Data are shown as values (95% confidence interval)**

**Supplemental Table 2**

Patient demographic, surgical, and radiographic data.

|  | **Valgus (n ＝ 33)** | **Varus (n ＝ 33)** | **P value** |
| --- | --- | --- | --- |
| **Patient demographics** |  |  |  |
| **Age (years)** | **59.7 ± 16.6** | **61.7 ± 7.0** | **0.53** |
| **Gender (male/female)** | **14/19** | **15/18** | **1.0** |
| **Height (cm)** | **160.9 ± 10.7** | **160.6 ± 7.1** | **0.89** |
| **Weight (kg)** | **66.7 ± 10.9** | **66.3 ± 8.6** | **0.86** |
| **BMI (kg/m²)** | **25.7 ± 3.1** | **25.7 ± 2.9** | **0.94** |
| **Radiographic evaluation** |  |  |  |
| **Preoperative FTA (°)** | **166.1 ± 7.7** | **180.3 ± 2.9** | **<0.01** |
| **Preoperative %MA (%)** | **88.9 ± 33.4** | **19.2 ± 14.9** | **<0.01** |

*n,* number of knees; *FTA,* femorotibial angle; *MA,* mechanical axis

Comparison between MCLA and LCLA in valgus and varus knees.

|  | **MCLA (°)** | **LCLA (°)** | **P value** |
| --- | --- | --- | --- |
| **Valgus** |  |  |  |
| **10 mm proximal** | **69.2 ± 8.7** | **76.3 ± 5.0** | **<0.01** |
| **Reference** | **67.2 ± 8.9** | **78.3 ± 4.4** | **<0.01** |
| **10 mm distal** | **66.1 ± 8.1** | **79.4 ± 4.6** | **<0.01** |
| **Varus** |  |  |  |
| **10 mm proximal** | **74.8 ± 6.5** | **81.0 ± 4.2** | **<0.01** |
| **Reference** | **74.6 ± 6.0** | **79.8 ± 5.2** | **<0.01** |
| **10 mm distal** | **72.1 ± 5.2** | **81.2 ± 4.4** | **<0.01** |

Values are the mean ± standard deviation.

**Comparison between MCH and LCH in valgus and varus knees.**

|  | **MCH (mm)** | **LCH (mm)** | **P value** |
| --- | --- | --- | --- |
| **Valgus** |  |  |  |
| **10 mm proximal** | **20.3 ± 3.6** | **26.9 ± 3.3** | **<0.01** |
| **Reference** | **21.3 ± 3.4** | **27.8 ± 3.6** | **<0.01** |
| **10 mm distal** | **23.6 ± 4.0** | **30.0 ± 4.3** | **<0.01** |
| **Varus** |  |  |  |
| **10 mm proximal** | **23.0 ± 2.8** | **28.6 ± 3.3** | **<0.01** |
| **Reference** | **24.2 ± 3.0** | **29.5 ± 3.0** | **<0.01** |
| **10 mm distal** | **26.5 ± 3.4** | **31.8 ± 3.4** | **<0.01** |

Values are the mean ± standard deviation.

**Comparison between valgus and varus knees in MCLA, LCLA, MCH, and LCH.**

|  | **Valgus** | **Varus** | **P value** |
| --- | --- | --- | --- |
| **MCLA (°)** |  |  |  |
| **10 mm proximal** | **69.2 ± 8.7** | **74.8 ± 6.5** | **<0.01** |
| **Reference** | **67.2 ± 8.9** | **74.6 ± 6.0** | **<0.01** |
| **10 mm distal** | **66.1 ± 8.1** | **72.1 ± 5.2** | **0.047** |
| **LCLA (°)** |  |  |  |
| **10 mm proximal** | **76.3 ± 5.0** | **81.0 ± 4.2** | **0.13** |
| **Reference** | **78.3 ± 4.4** | **79.8 ± 5.2** | **0.21** |
| **10 mm distal** | **79.4 ± 4.6** | **81.2 ± 4.4** | **0.12** |
| **MCH (mm)** |  |  |  |
| **10 mm proximal** | **20.3 ± 3.6** | **23.0 ± 2.8** | **<0.01** |
| **Reference** | **21.3 ± 3.4** | **24.2 ± 3.0** | **<0.01** |
| **10 mm distal** | **23.6 ± 4.0** | **26.5 ± 3.4** | **<0.01** |
| **LCH (mm)** |  |  |  |
| **10 mm proximal** | **26.9 ± 3.3** | **28.6 ± 3.3** | **0.047** |
| **Reference** | **27.8 ± 3.6** | **29.5 ± 3.0** | **0.06** |
| **10 mm distal** | **30.0 ± 4.3** | **31.8 ± 3.4** | **0.068** |

Values are the mean ± standard deviation.
